# The William F. Jenks Memorial Prize

**Published:** 1888-03

**Authors:** 


THE WILLIAM F. JENKS MEMORIAL PRIZE.—The first triennial prize, of $250, under the Deed of Trust of Mrs. William F. Jenks, will be awarded to the author of the best essay on "The

Diagnosis and Treatment of Extra-uterine Pregnancy." The prize is open for competition to the whole world, but the essay must be the production of a single person. The essay, which must be written in the English language, or, if in foreign language, accompanied by an English translation, should be sent to the College of Physicians of Philadelphia, Penn., U. S. A., addressed to Ellwood Wilson, M. D., Chairman of the William F. Jenks Prize Committee, before January 1, 1889. Each essay must be distinguished by a motto, and accompanied by a sealed envelope bearing the same motto, and containing the name and address of the writer. No envelope will be opened except that which accompanies the successful essay. The committee will return the unsuccessful essays if reclaimed by their respective writers, or their agents, within one year. The committee reserves the right to make no award if no essay submitted is considered worthy of the prize.

